# Experimental Study on Interface Bonding Performance of Frost-Damaged Concrete Reinforced with Yellow River Sedimentary Sand Engineered Cementitious Composites

**DOI:** 10.3390/ma18143278

**Published:** 2025-07-11

**Authors:** Binglin Tan, Ali Raza, Ge Zhang, Chengfang Yuan

**Affiliations:** 1 College of Civil Engineering, Zhengzhou University, Zhengzhou 450001, China; hayate3@126.com (B.T.); engrrazaa305@gmail.com (A.R.); 2 Yellow River Institute of Hydraulic Research, Yellow River Water Conservancy Commission, Zhengzhou 450003, China; gezhangyrihr@163.com; 3 Yellow River Laboratory, Zhengzhou University, Zhengzhou 450001, China

**Keywords:** ECC, Yellow River sedimentary sand, freeze–thaw cycle, interface bonding strength, bonding characteristics

## Abstract

Freeze–thaw damage is a critical durability challenge in cold climates that leads to surface spalling, cracking, and degradation of structural performance. In northern China, the severity of winter conditions further accelerates the degradation of concrete infrastructure. This study investigates the reinforcement of frost-damaged concrete using engineered cementitious composites (ECC) prepared with Yellow River sedimentary sand (YRS), employed as a 100% mass replacement for quartz sand to promote sustainability. The interface bonding performance of ECC-C40 specimens was evaluated by testing the impact of various surface roughness treatments, freeze–thaw cycles, and interface agents. A multi-factor predictive formula for determining interface bonding strength was created, and the bonding mechanism and model were examined through microscopic analysis. The results show that ECC made with YRS significantly improved the interface bonding performance of ECC-C40 specimens. Specimens treated with a cement expansion slurry as the interface agent and those subjected to the splitting method for surface roughness achieves the optimal reinforced condition, exhibited a 27.57%, 35.17%, 43.57%, and 42.92% increase in bonding strength compared to untreated specimens under 0, 50, 100, and 150 cycles, respectively. Microscopic analysis revealed a denser interfacial microstructure. Without an interface agent, the bond interface followed a dual-layer, three-zone model; with the interface agent, a three-layer, three-zone model was observed.

## 1. Introduction

In cold regions, frost damage poses a significant threat to the durability and safety of concrete structures. In northern China, the harsh winter conditions accelerate the deterioration of concrete infrastructure. Instead of opting for resource- and energy-intensive demolition and reconstruction, it is more aligned with sustainable development to assess frost-damaged concrete structures and apply targeted reinforcement and repair strategies based on engineering standards. This approach promotes resource conservation and environmental protection. Engineered cementitious composite (ECC) is an advanced construction material developed to overcome the brittleness typically associated with conventional concrete [1]. It has the ability to achieve a tensile strain greater than 3% at its ultimate limit [2], providing superior ductility compared to conventional concrete [3,4,5]. Its high impact resistance, seismic energy dissipation, and long-term durability have been validated by numerous studies [6,7,8,9,10,11], making ECC an ideal material for structural reinforcement applications. ECC has been continuously promoted and applied in the field of structural reinforcement [12,13,14,15,16]. Wang et al. investigated the coupling effects of freeze–thaw (FT) cycles with other degradation factors, such as chloride penetration, sulfate attack, and alkali-silica reaction, and showed that they significantly intensify concrete deterioration. The combination of these factors often results in more severe damage than that caused by any single factor [17,18].

Scholars have conducted numerous experimental studies on the mechanical properties, bonding, and durability of ECC-reinforced and repaired existing concrete structures. Lim et al. [19] showed that the ECC repair ability of concrete beams was stronger under the same geometric shape and load conditions. Compared to the control system of concrete or typical reinforced concrete repair materials, it has better ductility, stronger energy absorption, and better crack width control. Kim et al. [20] found that, when sprayed ECC was used as a repair material, the flexural bearing capacity and ductility of the repaired beam were greatly improved compared to the commercial pre-filled mortar repaired beam. Zhu and Wang et al. [21,22] found that, after being repaired with ECC, the pipeline’s bearing capacity was approximately four times higher than the original, and its impermeability was enhanced. Cheng et al. [23] carried out a systematic study on the bonding properties between fiber-reinforced concrete and existing concrete, discussing the basic mechanical properties of bonding shear and splitting tensile strength between fiber-reinforced concrete and existing concrete, as well as the frost resistance and impermeability durability of the bonding surface. Xu and Cai [24] found that the partial replacement of concrete cover with ECC (including tensile steel bars) can effectively prevent or delay the cracking of concrete cover caused by steel corrosion and improve the service life of concrete structures or components. Sahmaran et al. [25] found that the bonding strength was significantly improved when ECC was used as the covering material. Under compressive load, the bond strength of the ECC layer in the composite concrete column specimen is greater than that of the underlying substrate concrete, with a compressive strength of approximately 30 MPa. Cui Shuangshuang et al. [26] found that setting grooves on the bonding surface and using interface agents improved both dynamic shear performance and ductility. Wei [27] found that, with age, the splitting tensile strength of ECC concrete increased, and grooved interfaces further enhanced this strength, especially with C40 concrete. Wang Nan et al. [28] found that, under the same pouring conditions, the bond strength increases with the increase of the roughness and compressive strength of the existing concrete bond surface; the bonding strength of the bonding specimens with a wet saturated bonding surface is higher than that of the bonding specimens with a dry bonding surface. Tian [29] found that salt freeze–thaw cycles had a significant negative effect on ECC–concrete bonding, with lower ECC strength and smoother interfaces showing greater reductions in shear strength. Li Pingxian et al. [30] found that freeze–thaw cycles sharply decreased bond shear strength due to reduced concrete strength and damage to the bonding surface.

However, due to the high cost of quartz sand and the depletion of natural aggregate reserves, researchers have actively explored alternative materials, such as recycled fine aggregate and industrial by-products, for use in ECC to promote sustainability and reduce environmental impact. Yellow River sedimentary sand (YRS) has been identified as a potential replacement for traditional sand. The particles of Yellow River sedimentary sand are finer, and using them can not only reduce sand costs but can also help address the issue of excessive sediment deposition in the lower reaches of the Yellow River of China. More than 80% of the sand particles in YRS are smaller than 75 μm, and its main components, SiO_2_ and Al_2_O_3_, meet the material requirements for ECC. These properties makes YRS a viable substitute for natural quartz sand as a fine aggregate in the production of ECC. Many researchers have used YRS in ECC and UHPC. Yang [31] investigated YRS as a partial replacement for natural quartz sand in high-performance concrete, finding that YRS can be used at various replacement levels without compromising key mechanical properties. Yuan et al. [32] noted that fully replacing quartz sand with YRS slightly reduced compressive strength while retaining excellent tensile strain capacity and crack resistance. Raza et al. [33] found that a 25% replacement of quartz sand with YRS in ECC maintained 97.5% of its mass and 79.4% of its flexural strength after 300 freeze–thaw cycles, confirming its durability under harsh conditions. Raza et al. [34] investigated the integration of YRS into 3D-printed ECC, focusing on their microstructural and mechanical performance. The results showed that the incorporation of YRS enhanced the rheological properties of the 3DP-ECC.

## 2. Research Aims, Scope, and Novelty

Frost damage is a critical factor impacting the longevity and safety of concrete structures in cold regions, particularly in northern China. Sustainable and effective repair methods are essential to prolong the service life of such infrastructure. This study focuses on the reinforcement of frost-damaged concrete using ECC produced with YRS as a sustainable alternative to natural quartz sand.

The main aims of this study are as follows:

- Investigate the effectiveness of ECC made with 100% YRS as a repair material for frost-damaged concrete structures.
- Address environmental concerns and material scarcity by offering a sustainable alternative to quartz sand, while mitigating river sedimentation through the use of Yellow River sedimentary sand in ECC repair applications.
- Conduct splitting tensile tests to evaluate the interface bonding strength between the ECC repair layer and the existing concrete substrate under freeze–thaw cycles.
- Analyze the influence of interface agents, surface roughness treatments, and freeze–thaw cycles on the bond performance of repaired specimens.
- Develop a multi-factor equation for predicting interface bonding strength.
- Examine the bonding mechanisms at the microstructural level using SEM and MIP techniques.

This research involves the preparation of ECC using Yellow River sedimentary sand as the sole fine aggregate, followed by its application in repairing frost-damaged concrete specimens. Splitting tensile tests are carried out to assess bond strength under varying surface treatments and freeze–thaw conditions. This study also utilizes microscopic analytical methods to explore the interface characteristics and underlying bonding mechanisms.

The novelty of this research lies in the application of ECC produced entirely from Yellow River sedimentary sand for the repair of frost-damaged concrete, a sustainable solution that addresses both material scarcity and environmental challenges such as river sedimentation. Previous studies primarily investigated the mechanical and durability properties of YRS-ECC as a primary construction material, but this study is the first to systematically evaluate its effectiveness as a repair material for deteriorated concrete in cold regions. The research offers new insights into sustainable repair technologies, proposes a predictive model for interface bonding strength under realistic environmental conditions, and provides a comprehensive analysis of the microstructural properties at the repair interface.

## 3. Materials and Methods

### 3.1. Materials

**Figure 1:**
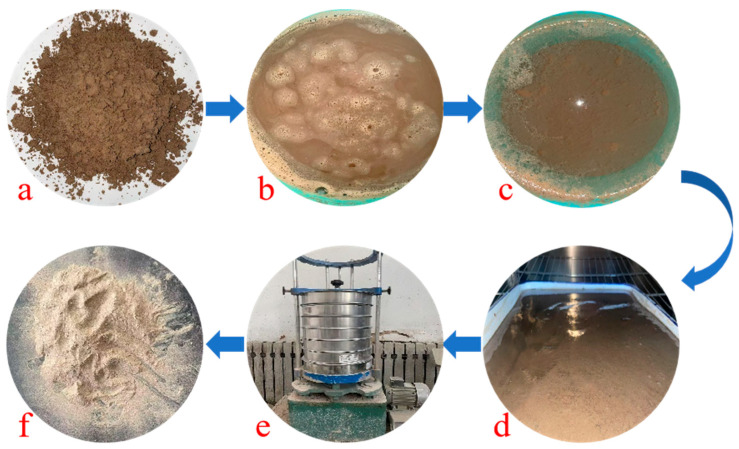
The pre-treatment process of Yellow River sedimentary sand: (**a**) original state, (**b**) cleaning, (**c**) filtration, (**d**) drying, (**e**) sieving, (**f**) finished product.

**Figure 2:**
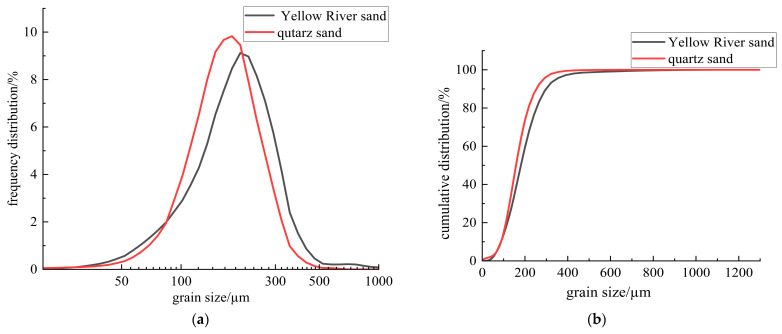
Particle size distribution of quartz sand and Yellow River sedimentary sand: (**a**) frequency distribution; (**b**) cumulative distribution.

**Figure 3:**
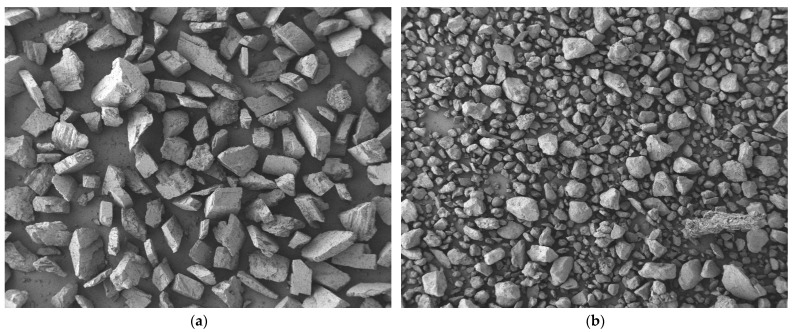
SEM micro-morphology (enlarged by 50 times): (**a**) quartz sand; (**b**) Yellow River sedimentary sand.

**Figure 4:**
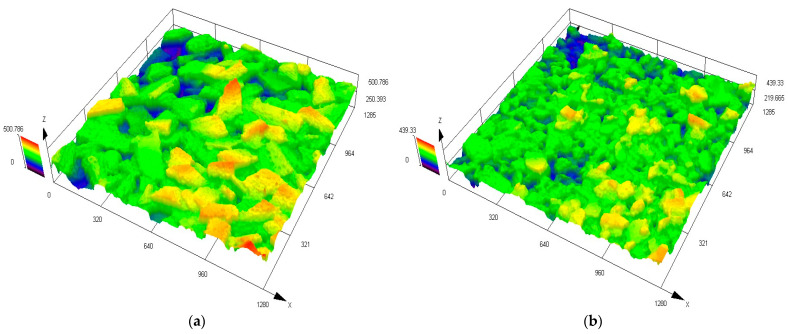
Three-dimensional topography images of the surface roughness: (**a**) quartz sand; (**b**) Yellow River sedimentary sand.

**Figure 5:**
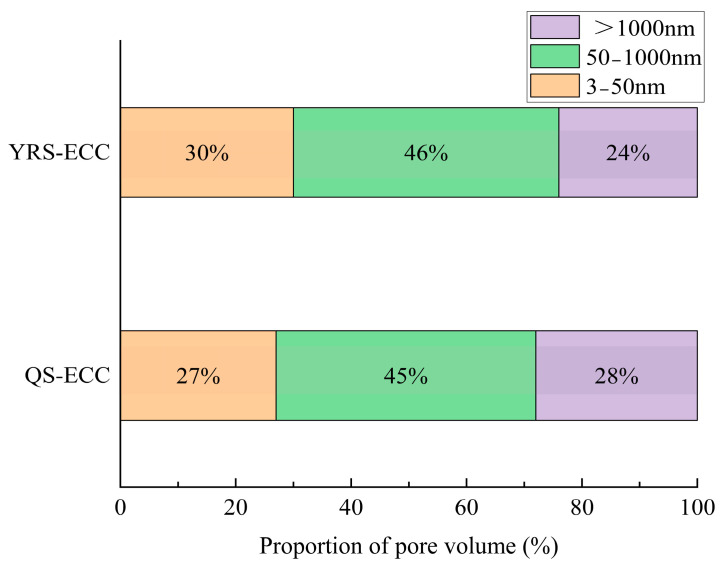
The proportion of pore volume of YRS-ECC and quartz sand ECC.

This study utilized P·O42.5 ordinary Portland cement produced by Zhengzhou Tianrui Cement Co., Ltd. of Henan Province, China to produce ECC, together with Grade II fly ash produced by Hengnuo Filter Material Co., Ltd. of Gongyi City, Henan Province, China as the supplementary materials. Monofilament polyvinyl alcohol fiber (PVA fiber) from Kuraray, Tokyo, Japan was selected for its reinforcing properties, with a length of 12 mm, a diameter of 40 μm, an elastic modulus of 41 GPa, a tensile strength of 1560 MPa, and an elongation at break of 6.5%. The admixture consisted of a high-performance polycarboxylate superplasticizer with a water reduction rate of 32% as a water reducer and HPMC-20 hydroxypropyl methylcellulose with a viscosity grade of 200,000 as a thickener, both produced by Shanghai Chenqi Chemical Technology Co., Ltd. (Shanghai, China). The fine aggregate is the pre-treated Yellow River sedimentary sand. The YRS used in the test was derived from the grit chamber of the Yellow River Diversion Project in Puyang City, Henan Province, China.The pre-treatment process (original state, cleaning, filtration, drying, sieving, finished product) is illustrated in Figure 1. The key technical parameters of the fine aggregate are outlined in Table 1, and the chemical composition is detailed in Table 2. The particle size distribution and SEM microscopic appearance of the YRS, magnified 50 times, is compared with quartz sand (80–120 mesh) in Figure 2 and Figure 3 [35,36]. Figure 4 displays the three-dimensional topography images of the surface roughness for both quartz sand and Yellow River sedimentary sand (YRS). The pore indices of Yellow River sedimentary sand ECC (YRS-ECC) and quartz sand ECC (QS-ECC), as measured by the MIP test, are presented in Table 3 and Figure 5.

Figure 3 shows that both quartz sand and YRS have irregular geometric surfaces with smooth textures. At the same magnification, YRS particles are finer than quartz sand, with a greater number of smaller-sized particles in the same volume. The shape of YRS particles is more rounded. This makes YRS a suitable replacement for quartz sand in ECC, as it can optimize particle gradation and improve slurry flowability.

Figure 4 shows the three-dimensional topography images of the surface roughness of quartz sand and YRS. In the test, the arithmetic mean height and root mean square height of the YRS are 36.385 μm and 45.968 μm respectively, both of which are lower than 45.618 μm and 57.368 μm of the quartz sand. Based on the images and key indicators, it is observed that the surface roughness of YRS is generally slightly lower than that of quartz sand, with particles that are smoother. This suggests that YRS has the potential to substitute quartz sand in the production of cement-based materials.

Table 3 and Figure 5 indicate that the porosity and average pore diameter of YRS-ECC are slightly smaller than those of QS-ECC, with similar pore size distribution ranges between the two. This suggests that using YRS in combination with cement to produce cement-based materials is a feasible option.

In preparing C40 concrete specimens, P·O 42.5 ordinary Portland cement was used. Natural river sand with a particle size of 0.25–0.5 mm served as the fine aggregate, while continuously graded crushed stone, with a particle size of 5–20 mm, was used as the coarse aggregate.

For the interface agent, P·O 42.5 ordinary Portland cement and YJ-302 interface agent were utilized, along with a UEA-type expansion agent with a 15-day longitudinal expansion rate of >0.02% and a 180-day longitudinal drying shrinkage rate of <0.02%.

### 3.2. Mix Proportion

By reviewing the literature and considering the specific engineering requirements [37,38,39], the volume content of polyvinyl alcohol (PVA) fiber was determined to be 1.5%. Yellow River sand (YRS) was utilized to completely replace quartz sand at a 100% mass substitution rate for the preparation of ECC. The resulting mix ratio for YRS ECC is detailed in Table 4.

Following the guidelines outlined in the JGJ 55-2011 [40], the mix ratio for C40 concrete was established and is presented in Table 5. The various types of interface agents employed are listed in Table 6.

### 3.3. Specimens Preparing

**Figure 6:**
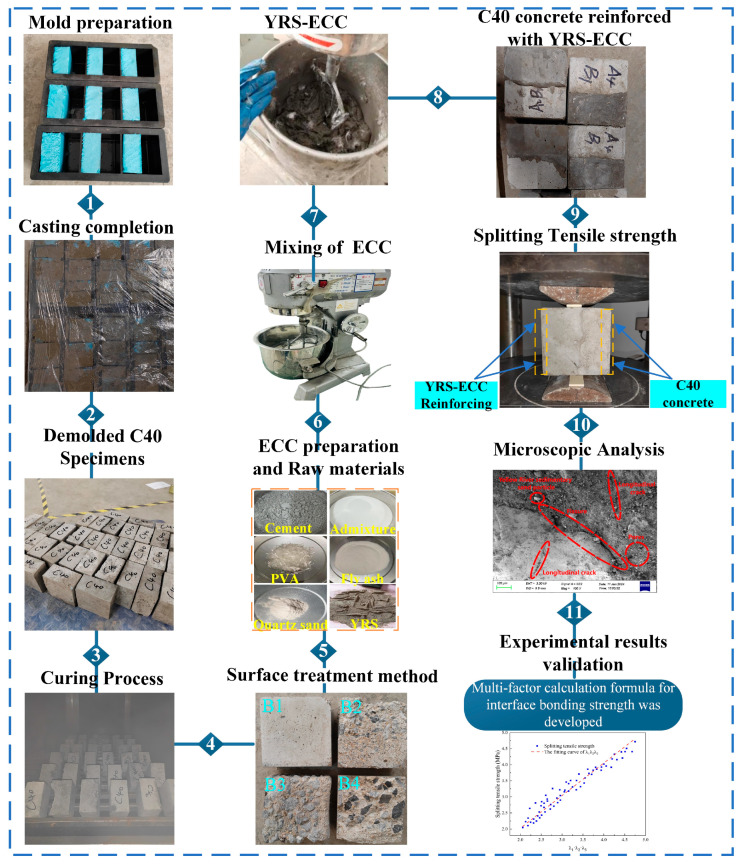
Complete research methodology and experimental process: mold preparation, 1. casting completion, 2. demolded C40 specimens, 3. curing process, 4. surface treatment method, 5. ECC preparation and Raw materials, 6. mixing of ECC, 7. YRS-ECC, 8. C40 concrete reinforced with YRS-ECC, 9. splitting tensile strength, 10. microscopic Analysis, 11. experimental results validation.

Three groups of C40 concrete specimens, each with dimensions of 100 mm × 100 mm × 50 mm, along with one group of 100 mm × 100 mm × 100 mm specimens, were cast. The production process for the former is outlined in Figure 6. The C40 concrete was initially subjected to 100 freeze–thaw cycles to simulate frost damage.

Afterward, the freeze-damaged C40 concrete underwent four different surface roughness treatments. Specifically, the ordinary C50 concrete specimens, each measuring 100 mm × 100 mm × 50 mm, were divided into three groups after the freeze–thaw cycles. The surface roughness treatments included untreated, manual chiseling, and mechanical chiseling, labeled as B1, B2, and B3, respectively. For the splitting tensile test, a 100 mm × 100 mm × 100 mm C40 concrete specimen was split after 100 freeze–thaw cycles, resulting in a 100 mm × 100 mm × 50 mm test block, referred to as the B4 interface.

Consequently, four distinct roughness interfaces were generated. The surface roughness of the damaged bonding interfaces was quantified using the sand-filling method, as outlined in reference [41]. The measured roughness for the four treatments yielded average sand-filling depths of 0.1–0.6 mm, 1.0–2.2 mm, 2.5–3.5 mm, and 3.5–4.5 mm, respectively. These surface profiles post-treatment are shown in Figure 6 (4). The C40 specimens, after surface roughness treatment, were submerged in water for 12 h, followed by drying and the application of interface agents. In this experiment, the following three types of interface agents were employed: cement slurry, expansive cement slurry, and YJ-302 bonding agent. The specimens were categorized according to the type of interface agent used, as follows: no interface agent (A1), cement slurry (A2), expansive cement slurry (A3), and YJ-302 agent (A4).

After the roughness treatment and application of the interface agent, the frost-damaged C40 concrete specimens were placed horizontally in custom wooden molds measuring 100 mm × 100 mm × 100 mm. YRS ECC with a 100% sand replacement ratio was poured on the opposite side of the mold. After an initial curing period of 48 h, the mold was removed, and the specimens underwent standard curing for 28 days [42]. This process resulted in the creation of reinforced ECC-C40 specimens, which were subsequently prepared for interface bond strength testing.

### 3.4. Experiment Method

In accordance with the standards outlined in GB/T 50082-2024 [43], a comprehensive testing design was implemented. The test specimens were all standardized to dimensions of 100 mm × 100 mm × 100 mm. The interface agents were categorized into the following four application methods: no interface agent (A1), coated with a cement net slurry interface agent (A2), coated with a cement expansion slurry interface agent (A3), and coated with a YJ-302 interface agent (A4). Additionally, the interface roughness treatment methods included no treatment (B1), the manual chiseling method (B2), the electric chiseling method (B3), and the splitting method (B4), resulting in four distinct interfaces. The specimens underwent freeze–thaw cycling with intervals set at 0, 50, 100, and 150 cycles. The interface bonding strength served as the primary evaluation index for assessing the interface bonding performance of ECC-C40-reinforced frost-damaged concrete. To minimize variability and enhance accuracy, three specimens were produced for each processing method for testing purposes.

According to GB/T 50081-2019 [44], the interface bonding strength test for the ECC-C40 specimens was carried out using the splitting tensile strength method. The test was performed with a YAW-2000B pressure testing machine and a prismatic steel block, as shown in Figure 6 (9).

The interface bond strength fts (MPa) of ECC-C40 specimens was obtained by experiments. On the basis of the splitting tensile strength value, the parameters Pn and Qn were introduced to better evaluate the interface bonding performance of ECC-C40 specimens. Pn is the splitting tensile strength ratio of ECC-C40 specimen and C40 concrete specimen after n freeze–thaw cycles, which reflects the reinforcement effect of YRS ECC on C40 concrete. Qn is the ratio of the ECC-C40 specimen to its own splitting tensile strength (0 freeze–thaw cycles) after n freeze–thaw cycles, which reflects the damage degree of the ECC-C40 specimen after freeze–thaw cycles. The specific formula is shown as follows:

(1) $$P_{n}=f_{tsn}^{EN}/f_{tsn}^{N}\times 100\%$$

(2) $$Q_{n}=f_{tsn}^{EN}/f_{ts0}^{EN}\times 100\%$$

In the formula, *f_tsn_^EN^* is the splitting tensile strength value of the ECC-C40 specimen under *n* freeze–thaw cycles (MPa); *f_tsn_^N^* is the splitting tensile strength of C40 concrete specimens under *n* freeze–thaw cycles (MPa); *f_ts__0_^EN^* is the splitting tensile strength value of the ECC-C40 specimen under 0 freeze–thaw cycles (MPa).

## 4. Test Results and Analysis

**Figure 7:**
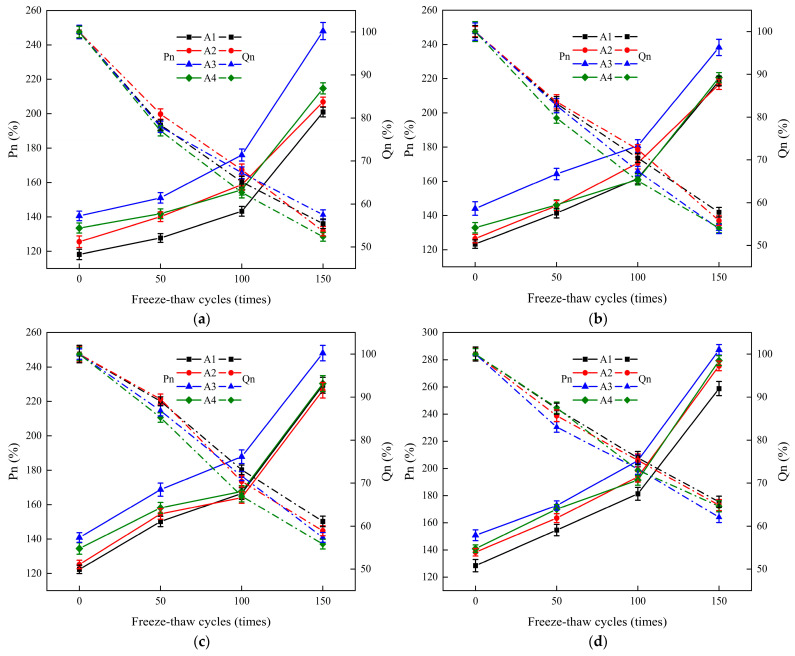
The effect of various interface agents on the interface bonding properties of ECC-C40 specimens subjected to four different roughness treatment methods: (**a**) no treatment (B1); (**b**) manual chiseling method (B2); (**c**) electric chiseling method (B3); (**d**) splitting method (B4).

**Figure 8:**
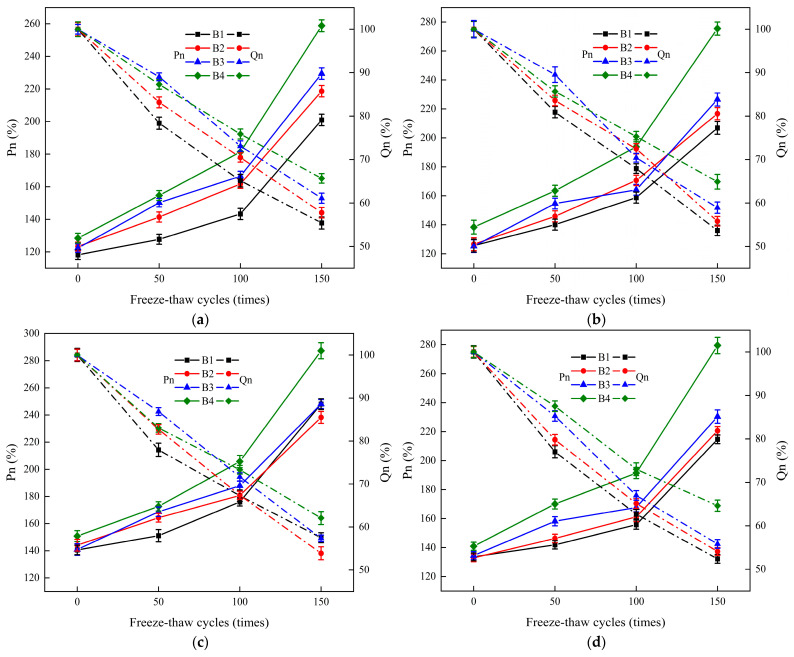
The influence of different interface roughness on the interface bonding properties of ECC-C40 specimens under four kinds of interface agents: (**a**) without interface agent (A1); (**b**) net cement slurry (A2); (**c**) expansion cement slurry (A3); (**d**) YJ-302 interface agent (A4).

The splitting tensile test was performed on the ECC-C40 specimens after undergoing freeze–thaw cycles. The splitting tensile strength values of the specimens were measured under varying interface agents, different surface roughness treatments, and after different freeze–thaw cycles. The corresponding Pn and Qn values were calculated. The impact of the interface agent and surface roughness on the splitting tensile strength of the specimens after freeze–thaw cycles was analyzed. The results are presented in Figure 7 and Figure 8.

Figure 7 illustrates that different interface agents significantly influence the bond strength (Pn) of the YRS ECC-C40 specimens. However, their effect on the durability index (Qn) is minimal. The bond strength (Pn) of the A3 group was the highest, while the A1 group exhibited the lowest Pn. A positive correlation was observed between Pn and the number of freeze–thaw cycles, with the B4 group achieving a maximum of 287.25% after 150 freeze–thaw cycles. This phenomenon occurs because, although the splitting tensile strength of both the ECC-C40 and C40 specimens decreases with an increasing number of freeze–thaw cycles, the strength of the freeze-damaged C40 concrete degrades at a faster rate. As a result, the ratio of the two Pn values increases, peaking after 150 freeze–thaw cycles.

During the freeze–thaw cycles, different interface agents had a minimal impact on the durability index (Qn) of the specimens. This is because Qn, which reflects the frost resistance and long-term durability of the concrete, is more influenced by the inherent material properties of the concrete, such as its aggregate composition, cement content, porosity, and hydration products, rather than the interface agents used. The Qn values were negatively correlated with the number of freeze–thaw cycles, showing similar degradation trends across all groups. After 150 freeze–thaw cycles, the B4 group had the highest Qn, exceeding 60%, while groups B1 to B3 basically ranged between 50% and 60%.

From 0 to 50 freeze–thaw cycles, the Qn for all specimens gradually decreased, with groups B1 and B2 experiencing a more rapid reduction. Between 50 and 100 freeze–thaw cycles, the rate of increase in Pn for B2 and B3 stabilized, while B1 and B4 began to accelerate. The Qn of B1, B2, and B4 decreased similarly, while B3′s rate of decline accelerated, with the most significant drop in A4B3. From 100 to 150 freeze–thaw cycles, all specimens showed a rapid increase in Pn and a gradual decrease in Qn. After 150 freeze–thaw cycles, the Pn values for specimens with different interface agents basically followed this order: A3 > A4 > A2 > A1, indicating that when the interface agent is a cement expansion slurry, the YRS ECC provides the most effective reinforcement against frost-damaged C40 concrete.

Figure 8 shows that different interface roughness treatments have a significant impact on the Pn of the interface bonding strength of the YRS ECC-C40 specimens, with the highest Pn value observed in the B4 group. The distribution basically follows the order B4 > B3 > B2 > B1, indicating that increased interface roughness continuously enhances the bonding performance of the reinforced specimens.

Regarding the number of freeze–thaw cycles, between 0 and 100 cycles, the Pn values increased slowly. After 100 freeze–thaw cycles, the maximum difference in Pn within the same group was between B4 and B1 in group A1, reaching 38.05%. From 100 to 150 freeze–thaw cycles, the Pn values of each group increased rapidly. After 150 freeze–thaw cycles, the maximum difference occurred between B4 and B1 in group A2, reaching 68.63%, which was significantly higher than at 100 cycles. In the later stages of freeze–thaw cycling, the B4 treatment showed considerable performance improvement, whereas C40 concrete rapidly declined in performance, albeit limited by the freeze–thaw effect. This resulted in a restricted Pn ratio, with the A2B4 group showing marked improvement.

During the later stages of the freeze–thaw cycles, the differences in Pn between groups with varying interface agents were under 20%. This is attributed to the C40 concrete having already undergone 100 freeze–thaw cycles. As the cycles continued, its performance worsened, and strength declined rapidly. When damage occurred on one side of the specimen, the ECC on the other side remained unaffected, thereby diminishing the effectiveness of both the interface agent and surface roughness treatments.

Throughout the freeze–thaw cycles, different interface roughness had little impact on the Qn of the specimens. Regardless of the interface agent treatment, the Qn decline trend was similar across all specimens, reaffirming that the interface agent has minimal effect on Qn. After 150 freeze–thaw cycles, the Qn values of the specimens decreased to between 50% and 65%, further validating the order B4 > B3 > B2 > B1. This indicates that the bonding performance of the specimens improves with increased surface roughness. Therefore, the YRS ECC demonstrated the best reinforcement effect on C40 frost-damaged concrete when the interface roughness was treated using the splitting method. The splitting tensile strengths of the specimens under the four interface agents were 28.78%, 33.17%, 15.81%, and 30.13% higher than that of the untreated group, respectively.

Overall, the optimal reinforcement effect is achieved when using cement expansion slurry as the interface agent and splitting method (B4) for interface roughness treatment. As shown in Table 7, under 0, 50, 100, and 150 freeze-thaw cycles, the splitting tensile strength of the reinforced specimens in Group A3B4 increased by 27.57%, 35.17%, 43.57%, and 42.92% respectively compared to those in Group A1B1. This indicates that when the number of freeze-thaw cycles is large, the performance improvement of reinforced specimens by applying interface agents and treating interface roughness is limited.

## 5. YRS ECC-C40 Interface Bonding Strength Calculation Method

Based on the analysis of the experimental results, an evaluation method for the interface bond strength between YRS ECC and C40 frost-damaged concrete is proposed, offering theoretical guidance for practical engineering applications.

Many scholars in China and abroad have put forward the calculation method of interface bond strength between new and old concrete. Gao [45] investigated the impact of different factors on the interface bond strength between two types of concrete and suggested an engineering evaluation method for the bond strength between lightweight aggregate concrete and conventional concrete. Wang [46] examined the influence of various factors on bond strength and developed a multi-factor calculation formula for the bond strength of ECC to existing concrete under axial tension, oblique shear, and direct shear conditions.

In this study, the effects of interface agents, interface roughness, and freeze–thaw cycles were analyzed, revealing that these factors have similar impacts on the interface bond strength of ECC-C40. Based on the research findings, the following multi-factor calculation formula for interface bond strength is proposed:

(3) $$\xi \left(f_{t}\right)=\alpha \lambda_{1}\lambda_{2}\lambda_{3}$$

In the formula, *ξ* is the interface bonding strength (MPa); α is the fitting coefficient (MPa); *λ*_1_ is the interface agent influence coefficient (dimensionless); *λ*_2_ is the roughness influence coefficient (dimensionless); *λ*_3_ is the freeze–thaw cycle number influence coefficient (dimensionless).

### 5.1. Determination of Influence Coefficient λ_1_ of Interface Agent

Based on the ratio of the splitting tensile strength values of the ECC-C40 specimens corresponding to the four interface agents, the influence coefficients of each interface agent were determined. When no interface agent is applied, the coefficient λ_1_ is 1.0000. For the cement slurry interface agent, λ_1_ is 1.0622. With the cement expansion slurry interface agent, λ_1_ equals 1.1892, and for the YJ-302 interface agent, λ_1_ is 1.1297.

### 5.2. Determination of Interface Roughness Influence Coefficient λ_2_

**Figure 9:**
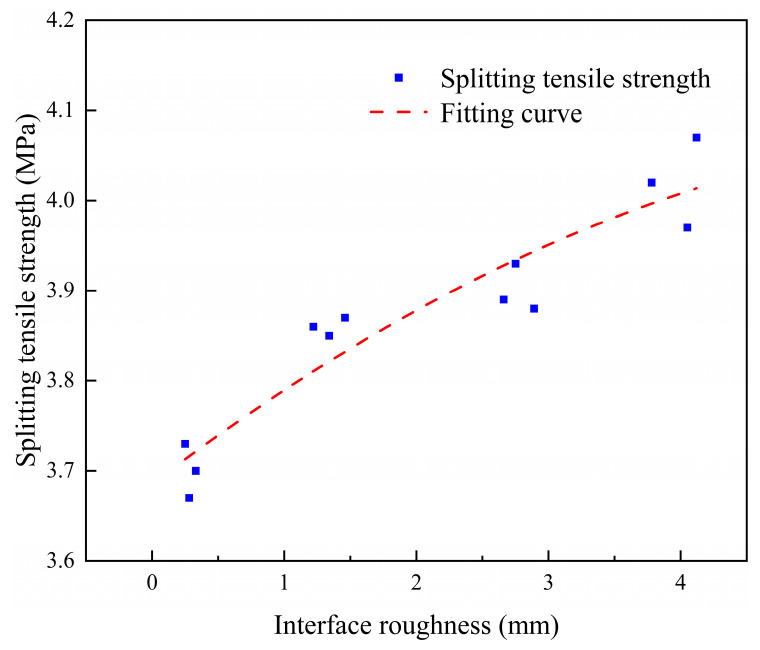
The relationship between interface roughness and splitting tensile strength.

From the experimental data obtained, the effect of interface roughness on the interface bond strength of ECC-C40 specimens was evaluated using a quadratic regression equation. The relationship between splitting tensile strength and roughness is represented by the equation y = −0.00789x^2^ + 0.1122x + 3.6851. The relationship curve is shown in Figure 9, R^2^ = 0.88875, and the fitting effect is good. The roughness of the interface (the average sand filling depth) is expressed by t [41], and the influence coefficient of interface roughness can be obtained using the following equation:

(4) $$\lambda_{2}=-0.00789t^{2}+0.1122t+3.6851$$

### 5.3. Determination of Freeze–Thaw Cycle Influence Coefficient λ_3_

**Figure 10:**
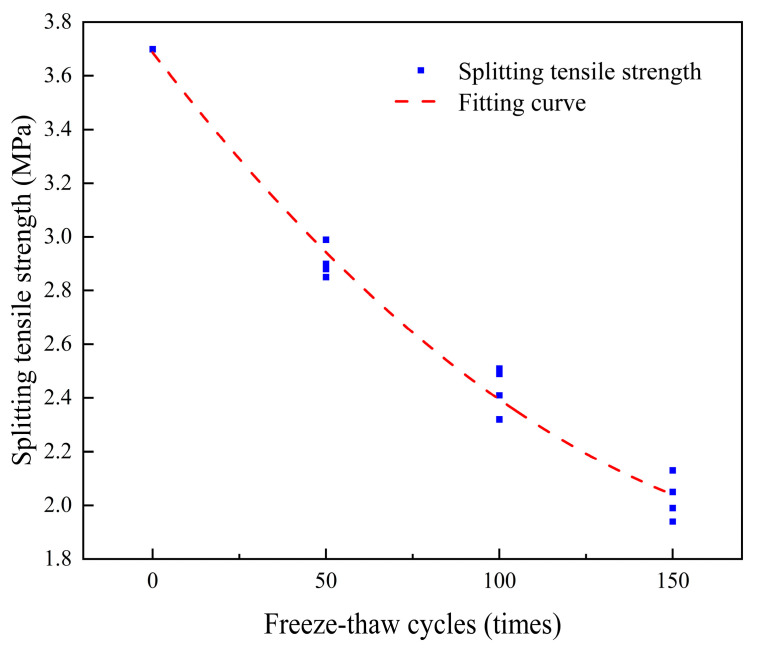
Relationship between freeze–thaw cycles and splitting tensile strength.

Using the quadratic regression equation to fit the test data, the relationship between the splitting tensile strength and the number of freeze–thaw cycles is *y* = 0.000039*x*^2^ − 0.01683*x* + 3.68725. The relationship curve is shown in Figure 10, R^2^ = 0.98936, and the fitting effect is excellent. Using n to represent the number of freeze–thaw cycles, the influence coefficient of the number of freeze–thaw cycles can be obtained.

(5) $$\lambda_{3}=3.9\times 10^{-5}n^{2}-0.01683n+3.68725$$

### 5.4. Determination of Fitting Coefficient α

**Figure 11:**
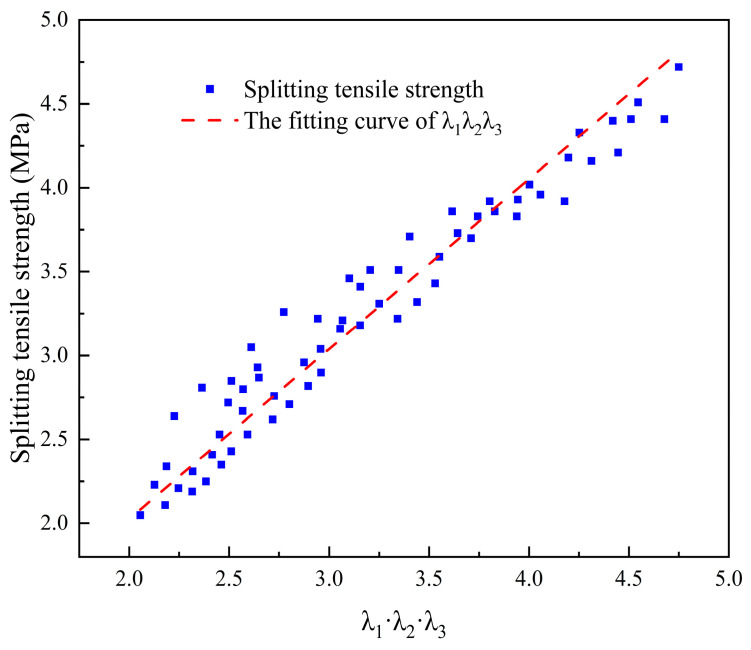
The relation curve between *λ*_1_·*λ*_2_·*λ*_3_ and splitting tensile strength *ξ*(*f_t_*).

The impact of various factors on the interfacial bond strength of ECC-C40 specimens was analyzed and modeled. The fitting coefficient α = curve slope *a* = 1.01316, as shown in Figure 11. The relationship curve is *y* = 1.01316x and R^2^ = 0.93348, indicating a good fitting effect.

Based on all the above data, the multi-factor calculation formula of the interface bonding strength of the YRS ECC-C40 specimen is shown as follows:

(6) $$\xi \left(f_{t}\right)=1.013\times (-0.00789t^{2}+0.112t+3.685)\times (3.9\times 10^{-5}n^{2}-0.0168n+3.687)\times \lambda_{1}$$

## 6. Analysis of Interface Bonding Mechanism and Model

To examine the surface bonding mechanism of the ECC-C40 specimen interface, SEM microscopic analysis was conducted to assess its microstructural properties. The interface between ECC and C40 concrete was scanned using an ultra-depth-of-field optical microscope, providing a detailed perspective on how the two materials interact at the interface. Based on these observations, a bonding model for the ECC-C40 interface was proposed.

### 6.1. Analysis of Interface Bonding Mechanism

**Figure 12:**
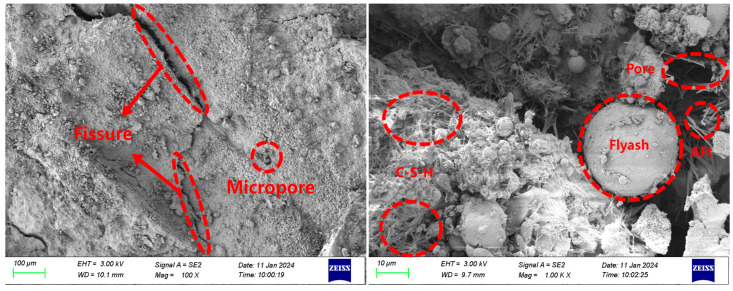
Micro-morphology of the bonding interface without an interface agent.

**Figure 13:**
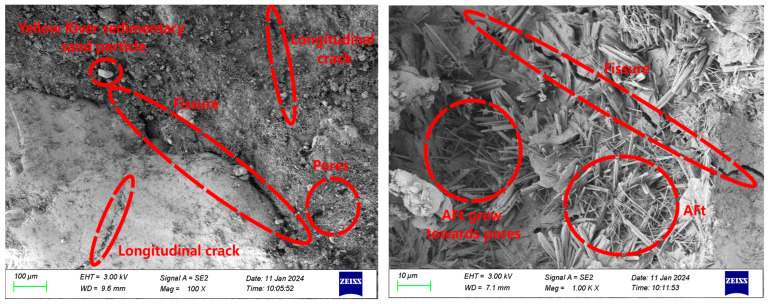
Micro-morphology of bonding interface with cement net slurry interface agent.

**Figure 14:**
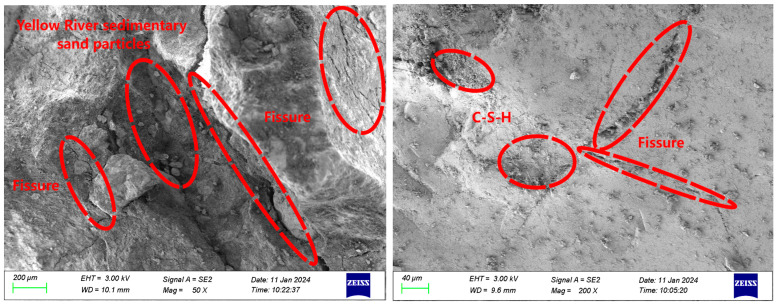
Micro-morphology of the bonding interface with a cement expansion slurry interface agent.

**Figure 15:**
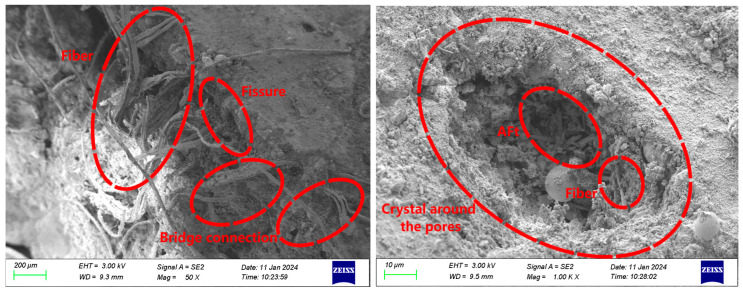
Micro-morphology of the bonding interface with the YJ-302 interface agent.

The SEM images illustrating the interface bonding of ECC-C40 specimens treated with four distinct interface agents are presented in Figure 12, Figure 13, Figure 14 and Figure 15.

Figure 12 shows the microscopic morphology of the bonding interface of ECC-C40 specimens without an interface agent. Clear cracks are observed in the bonding area between the ECC and C40 concrete. The YRS ECC exhibits a high degree of hydration, with little to no calcium hydroxide (Ca(OH)_2_) present. A limited amount of C-S-H gel and AFt crystals is formed near the bonding surface, but significant pores are observed on both sides, leading to weak interface compactness. This occurs because, without an interface agent, some ECC penetrates the pores of C40 concrete during long-term vibration, leading to the formation of additional micropores. This uneven cement distribution reduces the bonding area, increases cracks, and ultimately decreases the interface bonding strength.

Figure 13 shows that the microscopic morphology of the bonding interface for the ECC-C40 specimen treated with a cement slurry interface agent reveals that the cracks at the bonding interface between ECC and C40 concrete are narrower. Additionally, longitudinal cracks perpendicular to the main cracks are observed on both sides. More AFt crystals are observed growing into the pores. During the setting and hardening of the cement net slurry, Ca(OH)_2_ and AFt crystals form first upon contact with water. As hydration progresses, smaller C-S-H gel, Ca(OH)_2_ crystals, and AFt crystals from secondary crystallization fill the pores between the skeletons. The C-S-H gel forms an irregular network structure, which radiates outward toward the existing concrete during the bonding process and inward to tightly surround the cement particles, connecting them into a unified structure. This creates a tighter bonding interface.

Furthermore, since cement net slurry is a cement-based material with fluidity, it penetrates effectively into the rough surface of C40 concrete. The gel produced during hydration infiltrates the capillary pores of the C40 concrete, enhancing the mechanical interlocking at the bonding surface and improving interface bonding strength [46].

Figure 14 shows the microscopic morphology of the bonding interface for the ECC-C40 specimen treated with a cement expansion slurry interface agent. Near the bonding surface between ECC and C40 concrete, C-S-H gel forms, leading to a minimal presence of visible pores and a densely compacted bonding area. The UEA (ultra expansion agent) used in this study contains active salts, including aluminum sulfate and potassium aluminum sulfate. These salts promote the early formation of AFt crystals, which, combined with expansion effects, help compensate for shrinkage over a prolonged period [46]. After adding the expansive agent, the interface agent undergoes volume expansion, which is constrained by both the ECC and C40 concrete. This confinement allows more cement particles to come into contact with the surface of C40 concrete, generating preloading stress that enhances the mechanical interlocking between the aggregate and cement slurry, improving the interface’s tensile strength. AFt crystals grow radially into the pores of the structure, with fine crystals filling the capillary pores, reducing porosity, increasing density, and further improving the bonding strength at the interface.

Figure 15 illustrates the micro-morphology of the bonding interface of ECC-C40 specimens coated with the YJ-302 interface agent. Visible wide cracks are present on the bonding surface, yet a “bridging” effect occurs where PVA fibers from the ECC side extend to the C40 surface. This phenomenon enables the ECC and C40 concrete to remain bonded, despite the thinning of the interface agent during the pouring and vibrating process. As a result, numerous PVA fibers penetrate the interface agent and connect with the C40 concrete. Additionally, a substantial quantity of AFt crystals forms in the pores, and C-S-H crystals surround the holes, with some exhibiting a honeycomb-like structure. These crystals, fibers, and AFt clusters cover the surface of the structure, effectively filling capillary pores and reducing macropores. Consequently, the porosity decreases, resulting in a denser structure and enhanced interface bonding strength.

In general, compared to the A1 group of specimens without an interface agent, the materials on both sides of the three groups of specimens coated with an interface agent are clearer, and the structure is closer, which is consistent with the research of other scholars [47]. The existence of the interface agent enables the surface of C40 concrete to reach a moist saturation state, reduces bubbles and cracks at the interface, ensures a bonding area at the interface, and also increases the bite force between the interfaces. The cement particles in the interface agent can also enter the micro-voids on the surface of C40 concrete, making the C40 side structure denser, improving the performance of C40 concrete, and preventing the phenomenon of concrete crushing.

### 6.2. Analysis of Interface Bonding Model

One of the most widely accepted models for the bonding interface between old and new concrete is the three-zone model proposed by Emmnos et al. [48]. This model categorizes the bonding region into the following three distinct layers: the old concrete zone, the interface transition zone, and the new concrete zone.

Further research has expanded this concept by introducing a transition zone between cement paste and aggregate, consisting of the following three sub-layers: the diffusion interlayer, the strong effect interlayer, and the weak effect interlayer. In 1999, Li et al. [49] later adapted this theory, suggesting that the bond between new and old concrete follows a similar layered structure. In 2002, Zhao Zhifang [50] advanced the understanding by proposing a double interface-multilayer zone model, which describes the bonding layer as a composite of the new concrete zone, the old concrete zone, and a multi-layered transition zone. In this paper, the bonding interface of ECC-C40 was scanned using an ultra-depth-of-field optical microscope, and the microscopic morphology of the bonding interface was analyzed to establish an ECC-C40 bonding model.

**Figure 16:**
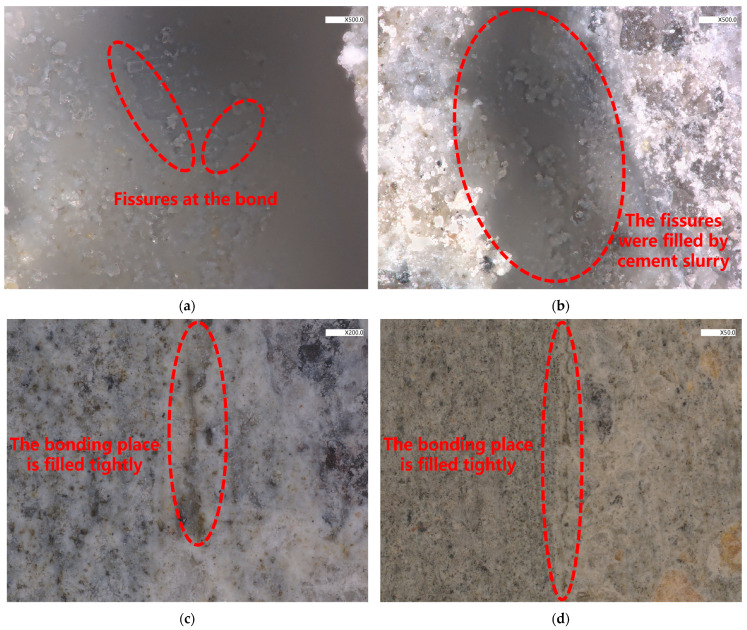
High-magnification microscope images of the ECC-C40 specimen interface bonding under different interface agents: (**a**) the interface bonding morphology of A1 (500× magnification); (**b**) the interface bonding morphology of A2 (500× magnification); (**c**) the interface bonding morphology of A3 (200× magnification); (**d**) the interface bonding morphology of A4 (50× magnification).

The damaged specimen’s bonding surface was analyzed using high-magnification optical microscopy, as illustrated in Figure 16. At 50× and 200× magnification, microcracks were observed both within the ordinary concrete near the interface and along the bonding surface itself. These cracks indicate reduced mechanical performance in the affected regions, suggesting that the transition layer closest to the concrete side is the weakest zone in ECC-C40. Consequently, stress-induced damage primarily initiates and propagates within this layer. Based on these findings, the bonding interface model for ECC-C40 (without an interface agent) aligns with the three-zone model, consisting of distinct layers with varying mechanical properties.

When the interface is not coated with an interface agent, a simple double-layer, three-zone model can be used to characterize the bond model of the specimen. This model comprises the corresponding C40 zone, the C40 and ECC transition zone, and the ECC zone, as illustrated in Figure 16a.

When an interface agent is applied to the bonding surface, its finite thickness creates a distinct affected zone. As a result, the conventional double-layer model must be modified into a three-layer system, comprising the old concrete, the interface agent layer, and the new concrete. The interface agent-affected zone itself consists of three key components, (1) a transition layer between the freeze-damaged C40 concrete and the interface agent, (2) varying interface agent zones depending on composition, and (3) a transition layer between the interface agent and the YRS ECC.

The formation mechanism of the old concrete interface agent transition layer is attributed to the higher water–binder ratio in this zone compared to the interface agent and ECC. This elevated ratio promotes the growth of AFt (ettringite) crystals, which dominate the microstructure of the transition layer and influence its mechanical behavior. Additionally, some active sodium ions and calcium ions from the interface agent and ECC penetrate into the pore structure of the old concrete, where they may react with the cement paste in the old concrete. This interaction is also a contributing factor to the formation of the transition layer. Given a certain type of interface agent and coating thickness, a higher porosity in the old concrete and a larger water–binder ratio in the ECC will result in a thicker transition layer between the old concrete and the interface agent.

The interface agent layer plays a critical role in bonding performance between ECC and ordinary concrete, typically maintaining a thickness of 2–3 mm. During the concrete placement process, the interaction between materials occurs through multiple mechanisms, such as when sodium and calcium ions from the ECC mixture migrate into the interface layer, while PVA fibers partially penetrate this transitional zone. This mutual exchange enables the interface agent to function effectively as a transitional medium during vibration, hydration, and subsequent chemical reactions. The bond formation mechanism between the interface agent and new concrete involves a gradual transformation where the freshly placed ECC develops into an integrated transition layer, exhibiting comparable crystalline structures throughout the affected zone. This integration is further enhanced by concurrent vibration and hydration processes, with the distributed PVA fibers in ECC providing additional reinforcement to the interfacial bond strength. The combined effect of these mechanisms results in improved mechanical performance at the transition zone between dissimilar concrete materials [46]. The interface agent effectively fills bonding surface cracks, creating the following three distinct zones visible under magnification: old concrete, interface transition layer, and new ECC. This microstructure confirms that the three-layer model accurately represents the ECC-C40 interface. The transition zone’s homogeneous composition, formed through material interpenetration during placement and curing, enables effective stress transfer across the interface. The model’s validation explains the observed improvement in bond performance compared to untreated specimens.

## 7. Conclusions

This study examines the interface bonding strength between engineered cementitious composites (ECC) and C40 concrete, with a particular focus on the effects of various interface agents and surface roughness. Through a series of freeze–thaw cycles, the performance of various interface agents was evaluated to understand their impact on bond strength and structural integrity. Microscopic analyses were conducted to investigate the bonding interface, providing insight into the mechanisms that influence bond formation and durability. The findings of this research aim to enhance the understanding of the bonding behavior between new and old concrete, contributing to the development of more effective reinforcement strategies in civil engineering applications.

(1) Different types of interface agents affect the interface bond strength of ECC-C40 specimens. After 150 freeze–thaw cycles, the bond strength of the four types of interface agents in the corresponding specimens follows the trend A3 > A4 > A2 > A1. This indicates that when the interface agent is A3, the YRS ECC exhibits the best reinforcement effect on C40 freeze-damaged concrete. Across four roughness levels, specimens without an interface agent showed 23.41%, 8.97%, 8.11%, and 10.98% higher splitting tensile strength than A1. This consistent improvement suggests enhanced mechanical interlock without bonding agents.

(2) Different interface roughness significantly influences the bond strength of ECC-C40 specimens. After 150 freeze–thaw cycles, the bond strength follows the trend B4 > B3 > B2 > B1. This indicates that bond strength increases with greater interface roughness, with specimens treated with B4 exhibiting the most effective reinforcement. Following 150 freeze–thaw cycles, B4 exhibited 28.78%, 33.17%, 15.81%, and 30.13% greater splitting tensile strength than B1 across the four interface treatments, demonstrating superior durability under thermal cycling conditions.

(3) The damage to ECC-C40 specimens from freeze–thaw cycles increases with the number of cycles. After 150 cycles, *Q_n_* values are between 50% and 70%, indicating some inhibition of bonding strength loss after reinforcement. The best reinforcement occurs with interface agent A3 and roughness B4, with the A3B4 group’s splitting tensile strength being 27.57%, 35.17%, 43.57%, and 42.92% higher than the A1B1 group under 0, 50, 100, and 150 cycles, respectively. These results indicate that, after extensive freeze–thaw cycling, both interface agents and surface roughness treatments provide diminishing returns for performance enhancement.

(4) The analysis presents a linear regression equation and a multi-factor computational model for ECC-C40 interfacial bond strength, both of which demonstrate strong fitting performance. These formulations provide valuable theoretical guidance for practical engineering design and implementation.

(5) Microscopic analysis revealed that ECC-C40 specimens without the interface agent exhibited large cracks and poor compactness, primarily due to insufficient cement hydration, resulting in minimal C-S-H gel and AFt crystal formation. In contrast, applying the interface agent significantly improved the interface compactness. This enhancement also resulted in enhanced bonding performance at the interface.

(6) The bonding interface of ECC-C40 specimens was analyzed using ultra-depth-of-field high-power optical microscopy. Specimens without an interface agent exhibited a bonding model characterized by ECC, a transition zone, and a double-zone model for C40. In contrast, those with an interface agent demonstrated a three-zone and three-layer bonding model, including the interface agent transition layer.

## Figures and Tables

**Table 1:** Main technical indexes of Yellow River sedimentary sand.

Apparent Density/kg·m^−3^	Bulk Density/kg·m^−3^	Water Absorption at Saturated Surface-Dry Basis/%	Specific Surface Area/m^2^·g^−1^
2647	1418	1.1	0.435

**Table 2:** Main chemical compositions of Yellow River sedimentary sands.

Component	SiO_2_	Al_2_O_3_	Fe_2_O_3_	CaO	K_2_O	Miscellaneous
Content/%	72.52	10.67	3.32	4.36	2.98	6.45

**Table 3:** The pore indices of ECC.

Constituencies	Porosity (%)	Average Pore Diameter (nm)	Pore Size Distribution Interval		
			3 nm~50 nm	50~1000 nm	>1000 nm
QS-ECC	32.78	36.03	27%	45%	28%
YRS-ECC	19.00	33.31	30%	46%	24%

**Table 4:** YRS ECC mix ratio (kg/m^3^).

Quartz Sand	Cement	Fly Ash	YRS	Water	PVA Fiber	Water Reducing Admixture	Thickening Agent
0	810	360	700	410	19	3.5	1.8

**Table 5:** Concrete mix proportion of C40 (kg/m^3^).

Strength Grade	Cement	River Sand	Crushed Stone	Water
C40	398	649	1155	199

**Table 6:** Types of interface agents.

Number	A4	A3	A2	A1
Constituencies	YJ-302 interface agent	Cement expansion slurry: bulking agent = 1:0.5:0.05)	Cement net slurry (cement:water = 1:0.5)	No interface agent

**Table 7:** The splitting tensile strengths (Mpa) of A3B4 and A1B1 under freeze-thaw cycles.

Freeze-Thaw Cycles	0	50	100	150
A1B1	3.70	2.90	2.41	2.05
A3B4	4.72	3.92	4.46	2.93

## Data Availability

The original contributions presented in this study are included in the article. Further inquiries can be directed to the corresponding author.
